# Ocular microtremor: a structured review

**DOI:** 10.1007/s00221-023-06691-w

**Published:** 2023-08-26

**Authors:** Lisa Graham, Julia Das, Rodrigo Vitorio, Claire McDonald, Richard Walker, Alan Godfrey, Rosie Morris, Samuel Stuart

**Affiliations:** 1 Department of Sport, Exercise and Rehabilitation, Northumbria University, Newcastle upon Tyne, UK; 2 Gateshead Health NHS Foundation Trust, Gateshead, UK; 3 Northumbria Healthcare NHS Foundation Trust, North Shields, UK; 4 Department of Computer and Information Science, Northumbria University, Newcastle upon Tyne, UK; 5 Department of Neurology, Oregon Health & Science University, Portland, OR USA

**Keywords:** Ocular microtremor, Eye movements, Biomarker, Neurological injury, Neurological disease

## Abstract

Ocular microtremor (OMT) is the smallest of three involuntary fixational micro eye movements, which has led to it being under researched in comparison. The link between OMT and brain function generates a strong rationale for further study as there is potential for its use as a biomarker in populations with neurological injury and disease. This structured review focused on populations previously studied, instrumentation used for measurement, commonly reported OMT outcomes, and recommendations concerning protocol design and future studies. Current methods of quantifying OMT will be reviewed to analyze their efficacy and efficiency and guide potential development and understanding of novel techniques. Electronic databases were systematically searched and compared with predetermined inclusion criteria. 216 articles were identified in the search and screened by two reviewers. 16 articles were included for review. Findings showed that piezoelectric probe is the most common method of measuring OMT, with fewer studies involving non-invasive approaches, such as contact lenses and laser imaging. OMT frequency was seen to be reduced during general anesthesia at loss of consciousness and in neurologically impaired participants when compared to healthy adults. We identified the need for a non-invasive technique for measuring OMT and highlight its potential in clinical applications as an objective biomarker for neurological assessments. We highlight the need for further research on the clinical validation of OMT to establish its potential to identify or predict a meaningful clinical or functional state, specifically, regarding accuracy, precision, and reliability of OMT.

## Introduction

Eye-tracking can provide insights into underlying cognitive mechanisms, such as attentional processing (Moran et al. 2018). Eye-tracking is utilized in various fields, such as medicine (Anderson and MacAskill 2013; Molitor et al. 2015), psychology (Hannula et al. 2010), sports (Discombe and Cotterill 2015; Kredel et al. 2017), and consumer behavior (Al-Azawai 2019; Bialkova et al. 2020). Examination of eye movements can provide an objective measure within neurological assessment, as specific eye movements can be attributed to specific brain regions and their functions (Stuart et al. 2019a; Hikosaka et al. 2000). Eye movements have been primarily categorized as saccades (rapid, jerk-like movements from one point of fixation to the next (Wade et al. 2003)), and fixations (time spent with eyes fixed on a visual target). Decline in ocular motor function has been linked to both neurological injury and disease, highlighting the potential in understanding, diagnosis, treatment, and prognosis of such impairments (Anderson and MacAskill 2013; Molitor et al. 2015; Mucha et al. 2014). Measurement of eye movements in neurological injury and disease is currently subjective, depending on clinical opinion providing potential for misdiagnosis (Baumann 2012; Tolosa et al. 2006). However, with advances in technology, objective measurement alternatives are becoming more readily available.

Ocular microtremor (OMT) may provide an objective measure for clinical use. First described in 1934, OMT is one of three involuntary eye movements that are present even when the eye appears still (Adler and Fliegelman 1934). OMT is considered a fixational movement and is a constant small amplitude, high-frequency tremor of both eyes (Robertson and Timmons 2007). OMT has been linked to the constant activity of the extra-ocular muscles stimulated by impulses from oculomotor neurons found in the brainstem (Robertson and Timmons 2007; Sheahan et al. 1993; Bolger et al. 1999; Shakhnovich 2012; Bojanic et al. 2001). Changes seen in OMT frequency occur because oculomotor neurons are embedded within the reticular formation of the brainstem (Robertson and Timmons 2007). The frequency of OMT ranges from 70 to 130 Hz in healthy individuals (Bolger et al. 1999a). Significantly lower OMT frequency has been recorded in patients under anesthesia (Bojanic et al. 2001) and a lack of OMT frequency has been recorded in individuals diagnosed with brainstem death (Bolger et al. 1999). Thus, OMT may provide a clinical tool for assessing brain stem function (Bolger et al. 1999a, 1999b; Coakley and Thomas 1977). Previous work has illustrated that OMT frequency changes with age, with significantly lower OMT frequency in those over the age of 60 (Bolger et al. 2001). Neurological diseases, such as Parkinson’s disease (PD) and multiple sclerosis (MS), have also shown decreased OMT frequency when compared to healthy controls (Bolger et al. 1999, 2000).

There is no gold standard technique for OMT measurement and methods range from invasive (e.g., piezoelectric techniques) to modern non-invasive technological assessment with eye-tracking devices (McCamy et al. 2013; Bengi and Thomas 1968; Kenny et al. 2013, 2014; Torre et al. 2016). Due to the lack of gold standard, the application of these OMT measurement protocols varies, which limits generalizability and interpretation of underlying deficits. Investigators who wish to measure and study OMT are left with a choice of measurement techniques and protocols that differ in many respects. In the process of developing robust and feasible protocols for clinical research, it is helpful to have evidence-based guidance. We therefore aimed to systematically review and summarize the current literature on OMT measurement and outcomes to aid the progression of future research.

We focused this review on the following: (1) the populations previously studied with OMT; (2) instrumentation used to measure OMT; (3) commonly reported OMT outcomes; and (4) recommendations concerning protocol design and future studies.

## Methods

### Search protocol

Four electronic databases were searched: PubMed, SCOPUS, Science Direct, and Web of Science. The key search terms in this review were “ocular microtremor” and “ocular micro tremor”. The search was limited to papers published between 1990 and April 2023 full journal articles only and articles written in English language to eliminate the potential for translational errors creating confusion due to complex language used. Studies were considered relevant if they incorporated terminology which contained the search term “ocular microtremor” in the title, abstract, or keywords. Findings were then screened, and any duplicates were removed. The remaining articles were then reviewed with consideration of the predetermined inclusion and exclusion criteria. These studies were then included in the review shown in Table 1.

**Table 1:** Inclusion and exclusion criteria

Inclusion criteria	Exclusion criteria
Published in the English Language	Publications in languages other than English
Human participants	Studies using non-human participants
Studies that objectively measure OMT either clinically or experimentally	Review papers
Studies using an observational design	Conference papers
Papers with full text available	Studies which have not been peer reviewed
	No access—i.e., papers were unable to be obtained through reasonable attempts via library and contacting the authors

### Inclusion and exclusion criteria

Table 2 provides inclusion and exclusion criteria for papers, alongside the rationale for each criterion. Papers were only included if they demonstrated an objective measurement of OMT.

**Table 2:** Study populations, characteristics, methods, and key findings

Author (Year)	Sample	Sample characteristics	Methods used	OMT characteristics	Key findings
Bolger et al. (1992)	*n = *8 (5 m, 3f), Mean age 34	Healthy, free from medication, no history of neurological trauma	PZT Participant lies supine with eyes fixating in the primary position Perspex rod mounted in a headframe and lowered directly onto the sclera with eyelids retracted with adhesive tape At least 30 s of OMT recorded from each subject	Recording time: 5–10 min OMT Frequency: HC = 84.99 Hz (SD = 8.6 Hz)	A relatively short record duration is very reliable to estimate overall frequency Authors suggest 5 s is the most convenient, but all showed at least 85% reliability
Sheahan et al. (1994)	*n = *12 (9 m, 3f), Aged 23–43	Healthy, no history of neurological trauma	PZT Participant lies supine with eyes fixating in the primary position Eye surface anesthetized and eyelids retracted using surgical retractors. Probe attached to a fixation device mounted on the head Probe lowered onto the eye surface and OMT frequency measured by counting peaks per 1 s. Ten such estimates were made for each record	Recording time: 30 s OMT Frequency: HC = NR Significant differences were observed for day-to-day variation in OMT frequency No significant differences were seen between the left and the right eye or between operators	Eyelid retractors caused 4 patients to withdraw sue to discomfort Of the remaining 118 records, 13 (11%) were rejected because the signal amplitude was too low to be seen clearly on the printed signal The set-up procedure contributes some variance to the measured OMT frequency No significant differences between measurements taken from left and right eyes Significant day-to-day variations within subjects were observed
Brown et al. 1998 (1998)	*N = *8 (4 m,4f), Aged 24–60	Clinically definite MS	Accelerometers Eyelids are gently taped shut and head movement restrained Accelerometer (0.5 mg) taped to eyelid at point of maximal convexity and 9 mm Electrodes taped to the inner and outer canthus of the eyes and one on the forehead Participant seated and instructed to make self-directed saccades between two light-emitting diodes arranged so as to be straight ahead of them They were asked to hold each position of gaze for ~ 4 s before the next saccade	Recording time: 30 min OMT Frequency: MS = NR HC = NR	OMT was more pronounced during saccade but returns to its former level after saccade OMT activity (peak to peak acceleration) was reduced by 85% in those with MS related internuclear compared to HC
Bolger et al. (1999)	*n = *6 (4f,2 m), Aged 33–63	Ocular motor palsy	PZT Participant lies supine with eyes fixating in the primary position Transducer mounted on a headset Probe lowered onto anesthetized scleral surface	Recording time: ~ 30 secs per eye OMT Frequency: Normal eye = 88.4 Hz SD ± 16.9 and in the Affected eye = 59 Hz SD ± 8.6 Hz No OMT was detected in the participant with the denervated eye	The authors suggest that innervation of the extraocular muscles is necessary for normal OMT activity, and OMT therefore has a neurogenic origin
Spauschus et al. (1999)	*n = *7 (6 m, 1f), Aged 29–47	Healthy	Two contact lens-mounted accelerometers (0.5 g) Both conjunctivae anesthetized and contact lens accelerometer placed shortly before experiment began Additional accelerometers placed on the forehead to account for any head movements Participants sat 57 cm from an oscilloscope and asked to perform self-paced saccades, smooth pursuit of a bright spot on the screen, passive, and active vestibular ocular reflexes Each task lasted 60–80 s	Recording time: ~ 5 Mins OMT Frequency: HC = extended up to almost 150 Hz, with peaks at low (0 to 25 Hz) and high (60–90 Hz) frequencies in both eyes	Conclude that synchronous and rhythmic discharge of extraocular motor units arises at a low level, probably within the brainstem
Bolger et al. (1999)	*n = *32 (17 m, 15f), Aged 18 – 87	Suspected brainstem death	Piezoelectric strain gage technique (PSG) Rubber tipped probe in a headset lowered onto anesthetized scleral surface	Recording time: OMT Frequency: HC = mean frequency of 89.6 Hz ± 6 Hz Comatose = mean frequency of 50.7 Hz ± 16 Hz (which was significantly lower than HC) BSD = no OMT activity in 28	In 3 patients who were not clinically diagnosed with BSD and showed OMT activity then went on to be diagnosed and OMT function was lost OMT is a sensitive method of detecting brain stem life and has the potential to play an important role in the assessment of BSD
Bolger et al. (1999)	*n = *105 (70 m, 35f), Aged 21–88	Healthy, No history of neurological/ocular/systemic disease. Aged > 21 years old	PSG	Recording time: OMT Frequency: HC = Mean peak count frequency of 83.68 Hz (SD = ± 5.78 Hz), Median peak count frequency = 83.8 Hz, Mode = 83 Hz, Range = 70 – 103 Hz	No significant difference between males and females Significant differences between those younger than 70 years old and those over 70 years old
Bolger et al. (1999)	*n = *44 (NS), Mean age 68 years	Diagnosis of Parkinson’s disease, no use of psychotropic or tranquilizing drugs. MMSE score > 28	PSG Rubber tipped probe lowered onto the anesthetized eye surface with the eyes in the primary position Eyelids retracted with adhesive tape Signal is amplified and stored on a Sony Walkman Both eyes were recorded independently	Recording time: 30 s OMT Frequency: HC = 81.64 Hz (SD = 6.10), Range = 72–91.8 Hz, Media*n = *83 Hz PD = 67.68 Hz (SD = 10.75), Range = 43–84 Hz, Media*n = *70.50 Hz Difference between PD and HC is significant PD OFF their medication were significantly lower than those who were ON (OFF = 58.88 Hz, SD = 10.35, O*N = *73.78 Hz, SD = 5.55 Hz)	One PD participant had withdrawn from medication for 48 h, and their frequency was 66.5 Hz but 24 h after taking their medication again, their frequency was 78.5 Hz The authors suggest that group studies (one ON the other OFF) may mask differences in the influence of dopaminergic medications due to between patient variation and so recommends that serial OMT readings per person would provide insight
Bolger et al. (2000)	*n = *53 (NS), Mean age 41.6 years	Diagnosis of MS/brainstem or cerebellar disease. Free from medication which could affect brainstem function. Ocular palsies	PSG Piezoelectric element mounted in a rubber tipped probe held in a head frame Probe was lowered onto the anesthetized scleral surface with eyelids retracted with tape Participant lay supine with eyes in the primary position Signal amplified and stored on a magnetic tape	Recording time: 30 s–60 s OMT Frequency: HC = 86.15 Hz (SD = 6.3) MS = 71.3 Hz (SD = 10.53) MS with brain stem disease = 67.09 Hz (SD = 8.9) MS without brain stem disease = 81.98 Hz (SD = 5.7)	The recording of ocular microtremor provides a new neurophysiological technique for the assessment of patients with multiple sclerosis
Bolger et al. (2001)	*n = *72 (NS), Mean age 54.22	Healthy. Free from medication. No history of neurological or ocular trauma. Aged > 21 Years	PZT Piezoelectric element mounted in a rubber tipped probe held in a head frame Probe was lowered onto the anesthetized scleral surface with eyelids retracted with tape Participant lay supine with eyes in the primary position Signal amplified and stored on a magnetic tape	Recording time: 30–60 s OMT Frequency: HC > 60 Years old = 80.5 Hz (SD = 4.7) HC < 60 Years old = 86.8 Hz (SD 5.5 Hz)	There was a significant drop in frequency for those 60 + years old Parameters of OMT were significantly correlated with age with the overall frequency being negatively correlated The strongest negative correlation was between frequency content of bursts and age
Bojanic et al. (2001)	*N = *22 (13 m, 11f), Aged 38–71	Undergoing surgery with propofol anesthesia	PSG Piezoelectric element mounted in a rubber tipped probe held in a head frame Probe was lowered onto the anesthetized scleral surface with eyelids retracted with tape Participant lay supine with eyes in the primary position Signal recorded on an audio cassette Recordings taken preoperatively (baseline) and during anesthesia	Recording time: 30 s–60 s OMT Frequency: Pre-propofol peak count frequency = 80.55 Hz (SD = 5.89 Hz) At loss of consciousness = 43.81 Hz (SD = 7.3) After loss of consciousness = remained below 55 Hz	One participant was abandoned for excessive coughing No significant difference was seen between loss of consciousness and readings after that at different propofol concentrations Authors suggested calculating a percentage baseline to predicts frequency at loss of consciousness At 45%, 17 of 21 subjects lost consciousness
Kevin et al. (2002)	*n = *30 (all male), Aged 17–59	Undergoing surgery with Sevoflurane anesthesia. ASA status 1 or 2. Free from CNS depressing medication. Aged > 18 and < 60 years. No history of ocular surgery	PSG and A-2000 BIS monitoring system BIS measured using A-2000 BIS monitoring system and Zepprep electrodes Piezoelectric element mounted in a rubber tipped probe held in a head frame Probe was lowered onto closed eyelid Signals amplified and displayed on oscilloscope Recordings taken preoperatively (baseline) and during anesthesia	Recording time: 1 min OMT Frequency: Awake = 85 Hz Unconscious = 48 Hz significantly decreased at loss of consciousness	BIS also decreased OMT significantly decreased at loss of consciousness OMT gave better logistical regression models for discriminating between awake and unconsciousness at the beginning of anesthesia OMT was better at discriminating between anesthesia and patient recovery The neuromuscular block used in ten patients caused decreased OMT amplitude, but it was still sufficient to measure
Heaney et al. (2004)	*n = *214 (NS), Aged 18 +	Undergoing intracranial or cardiothoracic surgery under Propofol or Sevoflurane anesthesia. Aged > 18 years. No history of ocular trauma or surgery	Closed eye PZT Piezoelectric element mounted in a rubber rod with a silicone tip Tip placed over closed eyelid and held in position by tape Signal amplified and displayed on oscilloscope	Recording time: 2.5 s epochs at 5 stages OMT Frequency: Awake = 68 Hz Unconscious = 40 Hz Maintenance 1 = 25 Hz Maintenance 2 = 35 Hz Emergence = 60 Hz	Mean OMT frequency significantly decreased at loss of consciousness and remained low until emergence OMT frequency did not change significantly in those patients turned from the supine to the prone position No difference between types of anesthesia and no sex differences were observed
McCamy et al. (2013)	*n = *8 (5 m, 3f), Age NS	Normal/normal to corrected vision	PZT (as described in previous studies included in review). OMT recordings were monocular EyeLink II video tracker helmet to record eye position non-invasively. Baseline eyelink was recorded prior to PZT application Participants fixated on a spot on a screen in front of them for one task Participants also performed the Troxler fading experiment–alerting the observer whether a target was fading/ intensifying	Recording time: Two 40 s trials were recorded for one or both eyes OMT Frequency: HC = NR	The piezoelectric technique effected microsaccades, but the video cannot see if OMT is affected The video system did not possess a high enough resolution to measure OMT Microsaccades and drift were treated as noise and removed from OMT readings by denoising to isolate the OMT frequency in the piezoelectric reading OMT frequency was not correlated with participant perceptual reports
Kenny et al. (2014)	*N = *20 (7 m, 13f), Aged 23–43	Healthy. no caffeine intake 2 h prior to testing	Laser speckle metrology HeNe laser within eye safe range, used with 10 s exposure is directed at the sclera EMCCD camera collects the reflected speckle images–500 frames/second Files then processed in Matlab Each speckle frame is cross correlated with the previous frame to measure displacement	Recording time: 10 s OMT Frequency: HC = Mean peak count was 78.27 ± 3.86 Hz	This paper highlighted the feasibility of a non-contact device for potential clinical use Confirmed that OMT can be measured using laser speckle Microsaccades cause noise but these can be removed in post processing If the participant blinked the reading was just taken again This paper calls for the need for a smaller, more portable device Piezoelectric methods require the participant to be in the supine position however this method can be carried out when seated
Ryle et al. (2015)	*n = *4 (all males), Aged 22–30	Healthy	Far field eye motion sensor Participant places head in headrests and fixates on a target slightly to the left Sclera illuminated by light source (4 different sources are tested) and two different imaging systems were used to capture Data is then digitally processed	Recording time: OMT Frequency: HC = inconclusive due to noise present in readings	OMT has different peak frequency components simultaneously in the vertical and horizontal directions This method does not require eyelids to be held open or a probe to be lowered onto the surface of the eye and so eye motions are not affected by mechanical loading

*PZT* Piezoelectric Transducer, *PSG* Piezoelectric Strain Gage, *OMT* ocular microtremor, *SD* standard deviation, *HC* healthy control, *PD* Parkinson’s Disease, *m* males, *f* female

### Data extraction and synthesis

Studies that met the inclusion and exclusion criteria were reviewed, and data pertaining to OMT were scrutinized by the reviewer (LG). Article titles and abstracts were also reviewed by a second reviewer (JD), and any discrepancies were resolved by an additional reviewer (RM). Data were extracted and synthesized into table format which were again confirmed by the second reviewer (JD). Key information was extracted from the articles, including demographics, sample size, control samples, and methods used. Study inclusion and exclusion criteria were also analyzed.

## Results

### Ocular microtremor data extraction

**Fig. 1:**
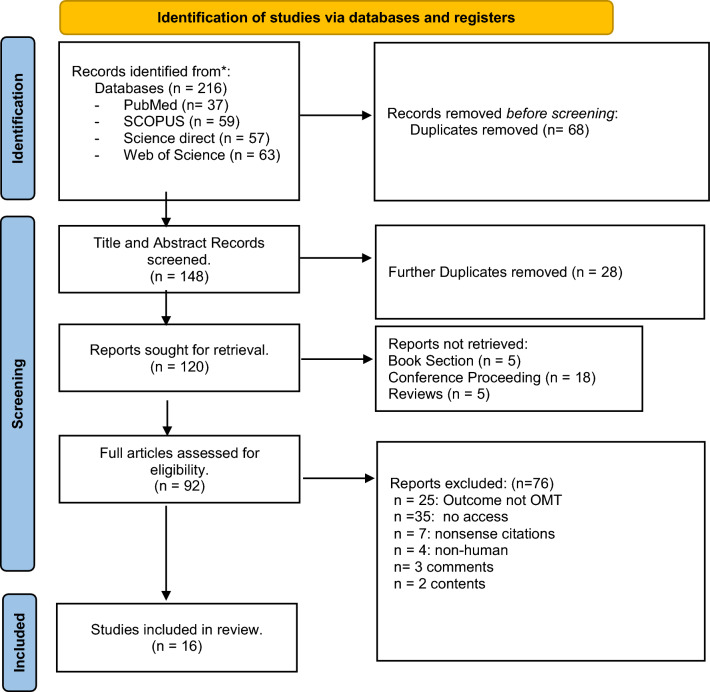
PRISMA Search Strategy. (Search updated April 2023) (Page et al. 2021)

Figure 1 provides a flow chart with information regarding the different phases of the search process. A total of 216 articles were obtained from the search, 68 duplicate articles were identified, and then removed leaving 148 to be screened via literature review software. A further 28 duplicates were removed, leaving 120 to be screened. Of the remaining articles, 35 were inaccessible. Where a paper could not be accessed, attempts were made to source the paper or contact the authors, but where no response was given, the paper was excluded from the review. Titles and abstracts of the remaining 92 articles were screened against the predetermined inclusion and exclusion criteria in the review software. Additional 25 were removed as OMT was not the outcome measure, four were removed for not using human participants, and seven were removed as they were nonsense citations. A further three were removed for being comments, and two contents removed. This left 16 articles which remained and underwent full-text screening. Articles included in the review are presented in Table 2.

### Sample populations

The reviewed articles (*n = *16) investigated healthy individuals (*n = *8) (Bolger et al.1999a, 1999b, 1992, Kenny et al. 2014; Sheahan et al. 1994; Spauschus et al. 1999; McCamy et al. 2013; Ryle et al. 2015), (Bolger et al. 1999), individuals scheduled for surgery under general anesthesia (propofol/sevoflurane) (*n = *3) (Bojanic et al. 2001; Kevin et al. 2002; Heaney et al. 2004), individuals with multiple sclerosis (*n = *2) (Brown 1998; Bolger et al. 2000), individuals with Parkinson’s Disease (*n = *1) (Bolger et al. 1999), suspected brain stem death (*n = *1) (Bolger et al. 1999), and oculomotor nerve palsy (*n = *1) (Bolger et al. 1999). Table 2 presents full details of all studies in chronological order. The sample size in the studies ranged from *n = *5 to *n = *214 participants (average *n = *40). The participants included in the reviewed studies were male dominant, (174 males out of 262 participants were reported–4 studies did not specify) with participants ranging from 17 to 88 years old.

### Inclusion and exclusion criteria

Inclusion and exclusion criteria were not specified in several of the studies (*n = *10). When they were included, history of neurological trauma or ocular disease was frequently identified as a source for exclusion (*n = *6), as was presence of medication which might affect brain stem function (*n = *2). Age was also identified as a limiting criteria (*n = *4) with two restricting the research to 18 + years old (Kevin et al. 2002; Heaney et al. 2004) and two studies to 21 + years (Bolger et al. 1999, 2001) due to concern regarding informed consent. Pertaining to concern with informed consent, a cognitive score on the mini-mental state examination (MMSE) of 28 + was required in one study (Bolger et al. 1999).

### Instruments and Procedures

Studies used a variety of techniques to measure OMT (see Table 2). Piezoelectric-based techniques were mostly used across the literature (*n = *12) (Bolger et al.^1992^;^1999^; 1999a;1999b;^2000^; Sheahan et al. 1994; Bojanic et al. 2001; McCamy et al. 2013), two of which involved closed eye recordings (Kevin et al. 2002; Heaney et al. 2004). Other methods, such as contact lens-mounted accelerometers (*n = *1) (Spauschus et al. 1999), non-contact far field method (*n = *1) (Ryle et al. 2015), eyelid mounted accelerometers (*n = *1) (Brown 1998), and laser speckle metrology (*n = *1) (Kenny et al. 2014), were also utilized.

Table 2 shows equipment utilized to measure OMT in the literature. Most investigators administered local anesthetic for the scleral surface (*n = *11). These were all piezoelectric studies apart from one which used contact lens-mounted accelerometers (Spauschus et al. 1999). To hold eyelids open, surgical tape was frequently used (*n = *10). Eye retractors were also reported (*n = *1). In some studies, head movement was anticipated as a source of noise and so to eliminate this, a headrest or a bite bar was used (*n = *5). Recordings were taken over various time periods with some studies taking serial readings and some taking continuous recordings for up to 1 min. Where reported, OMT measurement was collected monocularly. If both eyes were tested, this was done independently.

### Outcome measure: OMT characteristics

In all studies, the only reported outcome measure was OMT frequency. In healthy participants, the reported frequency ranged from 78.27 Hz (± 3.9 Hz) (Kenny et al. 2014) to 89.6 Hz (± 6 Hz) (Bolger et al. 1999b). OMT frequency was also observed in conscious and unconscious populations with the use of anesthesia. In the conscious, awake states, OMT frequency was reported as 80.6 Hz (SD = 5.9 Hz) (Bojanic et al. 2001) and 85 Hz (Kevin et al. 2002). This significantly reduced at the point of loss of consciousness to 43.8 Hz (SD = 7.3) (Bojanic et al. 2001) and 48 Hz (Kevin et al. 2002). Reviewed studies that looked at neurologically impaired populations reported OMT frequency was significantly reduced when compared to healthy individuals. In MS, OMT frequency was reported to be 71.3 Hz (SD = 10.5 Hz) and in those with MS and brain stem or cerebellar disease, 67.1 Hz (SD = 8.9 Hz) (Bolger et al. 2000). In PD, OMT frequency was 67.68 Hz (SD = 10.75) and in one participant, OMT was measured ON and OFF medication. There was a considerable difference between ON and OFF states with OMT frequency with ON being 73.8 Hz (SD = 5.6 Hz) which dropped to 58.9 Hz (SD = 10.6 Hz) when OFF (Bolger et al. 1999). It was also reported that there is no difference between OMT frequencies for each eye. However, frequency varied when measured on different days (Sheahan et al. 1994). In a sample of individuals with ocular nerve palsy, the normal, unaffected eye possessed an OMT frequency of 84.4 Hz (SD = 16.9), whereas the affected eye possessed a significantly lower frequency of 59 Hz (SD = 8.6) (Bolger et al. 1999). In another study, a significant negative correlation exists between OMT frequency and age (Bolger et al. 2001).

## Discussion

To the authors’ knowledge, this review presents the first systematic synthesis of the literature examining OMT. A total of 16 studies met the inclusion criteria and were examined based on their methods, participants, and findings. We focused this review on the following: (1) the populations previously studied with OMT; (2) instrumentation used to measure OMT; (3) commonly reported OMT outcomes; and (4) recommendations concerning protocol design and future studies. Current methods of quantifying OMT were reviewed to analyze the efficiency and reliability of OMT as a measure. This review found that OMT frequency differs between different populations and possesses major clinical potential. However, the current methodologies are highly invasive and limit the clinical applicability of OMT measurement.

### Sample populations

Sample size hugely varied across the reviewed studies (i.e., *n = *8–214). Reviewed studies with larger samples involved healthy participants, those under general anesthesia or brainstem death. Smaller sample sizes were seen for neurological cohorts, such as PD and MS, which will impact reported data and reduce results generalizability. PD and MS are heterogeneous conditions and therefore larger sample sizes are required to reflect the populations adequately. For example, some participants experienced blepharospasm (a painful spasm of the eyelids) when the eyes were held open with tape or instruments (Sheahan et al. 1994). No visual function or ability features were used for inclusion or exclusion criteria, and many studies did not commonly collect demographic data or information regarding habits/activities (e.g., hours of sleep, screen time, etc.). These may account for intra-subject variability and would be interesting to investigate in future work. Other eye-tracking studies typically require visual function to be normal or near normal to ensure accurate performance that is not impacted by visual function impairment (Stuart et al. 2019). While OMT should be present in all individuals, regardless of visual function, it may influence ability to follow instructions that involve visual function (i.e., look ahead at visual target). Generally, outcomes from small cohorts may not accurately reflect or represent that of the general population of interest and furthermore create a lack of statistical power that could lead to inconsistency in findings. Therefore, more work is required in PD, MS, and other clinical populations to study the efficacy of this outcome.

### Instruments and procedures

There was no consistent method of OMT measurement, but most reviewed studies used the piezoelectric method. This, however, possesses low clinical accessibility. First described by Bengi and Thomas in 1968 (Bengi and Thomas 1968), the piezoelectric technique for measuring OMT involves lowering a rubber probe onto the scleral surface while the eye is held open with an eyelid retractor or surgical tape (Bolger et al. 1999, 1999b). The piezoelectric element acts as a transducer and measures scleral displacement (Sheahan et al. 1994). While it is an accurate measure, it is outdated, and the invasive limitations outweigh the benefits, i.e., the need for the eye to be anesthetized (Bolger et al. 1999, 1992, 2000, 1999; Sheahan et al. 1994; Spauschus et al. 1999; Bojanic et al. 2001), inter-observer variability, requirement of a highly trained operator, and the fact that recording time is limited to reduce discomfort and eye dryness (Sheahan et al. 1994). Overall, this method is limited as an objective biomarker in clinical populations. Another potentially limiting factor of the piezoelectric method is the fact that it relies on constant uninterrupted contact with the sclera and there is an unknown loading effect of the probe. More recently, Ryle et al. (Ryle et al. 2015) demonstrated that a non-contact method was able to record OMT without causing a mechanical loading effect. This non-contact far field method described (Ryle et al. 2015) utilizes spatially incoherent illumination from a light-emitting diode (LED) and an ultra-fast, high-resolution black and white camera to capture eye movements. This method allows both horizontal and vertical fixational motions to be captured simultaneously without coming into contact with the eye itself. The relative frame-to-frame displacement could be calculated without having to tape eyelids open or probe the eyes surface. It is obvious that the piezoelectric method is outdated and is not clinically viable, thus limiting OMT as a potential clinical biomarker for neurological function or impairment.

A less-invasive technique described in the reviewed articles was the use of accelerometers, both contact lens-mounted accelerometers (Spauschus et al. 1999) and eyelid mounted accelerometers (Brown 1998). These techniques enabled changes in eye acceleration to be recorded during eye movements. The accelerometers weighed 0.5 g each and therefore are much more tolerable than the piezoelectric probe and having eyes held open. The accelerometer technique has a response range of up to 500 Hz—well above the expected range for OMT—and the response to acceleration in a plane other than the target direction is less than 3% suggesting that there is little noise interference (Brown and Day 1997). An additional accelerometer was also placed on the forehead to eliminate noise from head movements. This method allowed simultaneous recordings of both eyes. However, results in the reviewed articles showed little to no discrepancies between the two eyes in healthy subjects (Sheahan et al. 1994). Ratliff & Riggs (Ratliff and Riggs 1950) suggest that the reason this method did not gain widespread clinical acceptance is that they may not adhere to the eye adequately. However, this research was carried out in 1950 and as a result is likely outdated.

To ensure OMT provides an objective and clinically accessible biomarker, a non-invasive technique is required. In addition to the method used by Ryle et al. (2015), Kenny et al. (2014) also demonstrated that OMT could also be accurately measured non-invasively using laser speckle metrology (Kenny et al. 2014). Laser speckle metrology is the most recent method presented, and uses laser technology to create a speckle pattern of light and dark spots caused by interference (Kenny et al. 2013). This method offers a high-resolution non-invasive, compact, and portable technique for obtaining OMT data. It has been proven to be feasible and overcomes many of the current limitations associated with other more-invasive techniques (Al-Kalbani et al. 2009). This solution shows promise for non-invasive measurement of OMT; however, there is a need for more evidence using non-invasive devices for objective OMT measurement to provide clear understanding and allow accurate interpretation for clinical use.

### Outcome measure: OMT characteristics and implications

This review has found that OMT frequency differs between healthy individuals and clinical groups, specifically those under anesthesia, brain stem death, and those with neurological impairments (PD, MS). Overall, regardless of group the OMT frequencies range between 70 and 150 Hz, in line with previous reports (Bolger et al. 1999). OMT frequency was reduced compared to healthy controls in those under anesthesia and was not present in brain stem death. Similarly, OMT frequency was found to be able to differentiate neurological populations from healthy controls, which is similar to wider eye-tracking literature. Eye movements are controlled by the extraocular muscles, which interconnect via a tract in the brainstem (Bae et al. 2013). Basal ganglia (BG) and cerebellum modulate the neural system for saccade generation, duration, and amplitude. Considering such a substantial area of the brain is involved in eye movements, it is no surprise that abnormalities in eye movements are useful for understanding brain activity and neurological disorders (Lal and Truong 2019). This is highlighted by Bolger et al. (1999) (Bolger et al. 1999) whose work in ocular palsy supports the notion that OMT has a neurogenic origin. The drop in OMT frequency also seen with anesthesia at loss of consciousness is particularly interesting in highlighting the link between OMT and neural activity as it is essential for patients to be unconscious during surgery, so a reduction in brain activity corroborates this. Moreover, eye-tracking is invaluable in enabling clinicians to discern brain dysfunction from patterns of abnormality and attributes this to different neurological states or neurological disorders (Terao et al. 2017). This knowledge signifies the importance of research on OMT and its possibilities. Specifically, OMT is a constant, involuntary eye movement, so its characteristics could provide insights into neurological functions without the need for extensive eye-tracking or cognitive tests that require relatively intact ability to follow instructions (which is lost with neurodegeneration or neurological impairment). Based on the findings in this review, Table 3 highlights recommendations for future research into OMT.

**Table 3:** Research recommendations

Research recommendations
Sample size needs to be justified and adequate for statistical analysis (i.e., > 30 per group)
OMT should ideally be collected via non-invasive methods
OMT measurement should be performed while sitting or lying down
OMT Frequency is the most reported outcome, so should be included in future studies
OMT measurement time needs to be reported (e.g., 5 s)
Both eyes should be tested for OMT, unless clinical condition does not allow this
Finally, to confirm the validity of OMT as a clinical measure, future research should assess reliability

## Conclusion

This is the first review of OMT measurement that has shown that protocols vary between studies and that very few studies have examined clinical cohorts. OMT is a quantifiable involuntary eye tremor that can be measured with various technologies, with more recent devices being non-invasive. OMT is generally reduced in those under anesthesia, or with neurological conditions, and is absent in those with brain stem death. Further quantification of OMT is needed to determine the effect of specific clinical conditions on its frequency, and aid in the development of further OMT outcome measures. Moreover, further research on the clinical validation of OMT is required to establish its potential to acceptably identify or predict a meaningful clinical or functional state, specifically regarding accuracy, precision, reliability, and validity of OMT.

## Data Availability

This is a review article of already available, published data. The datasets generated during and/or analyzed during the current study are available from the corresponding author on reasonable request.

## References

[CR1] Adler FH, Fliegelman M (1934). Influence of fixation on the visual acuity. Arch Ophthalmol.

[CR2] Al-Azawai M (2019). The application of eye-tracking in consumer behaviour. Int J Eng Technol..

[CR3] Al-Kalbani M, Mihaylova E, Collins N, Toal V, Coakley D, Boyle G (2009). Ocular microtremor laser speckle metrology.

[CR4] Anderson TJ, MacAskill MR (2013). Eye movements in patients with neurodegenerative disorders. Nat Rev Neurol.

[CR5] Bae YJ, Kim JH, Choi BS, Jung C, Kim E (2013). Brainstem pathways for horizontal eye movement: pathologic correlation with MR imaging. Radiographics.

[CR6] Baumann CR (2012). Epidemiology, diagnosis and differential diagnosis in Parkinson's disease tremor. Park Rel Dis..

[CR7] Bengi H, Thomas JG (1968). Three electronic methods for recording ocular tremor. Med Bio Eng..

[CR8] Bialkova S, Grunert KG, van Trijp H (2020). From desktop to supermarket shelf: Eye-tracking exploration on consumer attention and choice. Food Qual Pref.

[CR9] Bojanic S, Simpson T, Bolger C (2001). Ocular microtremor: a tool for measuring depth of anaesthesia?. Br J Anaesth.

[CR10] Bojanic S, Simpson T, Bolger C (2001). Ocular microtremor: a tool for measuring depth of anaesthesia?. Article Brit J Anaesth.

[CR11] Bolger C, Sheahan N, Coakley D, Malone J (1992). High frequency eye tremor: reliability of measurement. Clin Phys Physiol Meas.

[CR12] Bolger C, Bojanic S, Sheahan NF, Coakley D, Malone JF (1999). Ocular microtremor in oculomotor palsy. J Neuroophthalmol.

[CR13] Bolger C, Bojanic S, Sheahan NF, Coakley D, Malone JF (1999). Dominant frequency content of ocular microtremor from normal subjects. Vision Res.

[CR14] Bolger C, Phillips J, Bojanic S, Sheahan N, Coakley D, James M (1999). Ocular microtremor in brain stem death. Neurosurgery.

[CR15] Bolger C, Bojanic S, Sheahan NF, Coakley D, Malone JF (1999). Ocular microtremor in patients with idiopathic Parkinson's disease. J Neurol Neurosurg Psychiatry.

[CR16] Bolger C, Bojanic S, Sheahan NF, Coakley D, Malone JF (1999). Ocular microtremor in patients with idiopathic Parkinson's disease. Art. J Neurol Neurosurg Psychiat..

[CR17] Bolger C, Bojanic S, Sheahan NF, Coakley D, Malone JF (1999). Dominant frequency content of ocular microtremor from normal subjects. Article Vision Res.

[CR18] Bolger C, Bojanic S, Phillips J, Sheahan N, Coakley D, Malone J (1999). Ocular microtremor in brain stem death. Article Neurosurg.

[CR19] Bolger C, Bojanic S, Sheahan N, Malone J, Hutchinson M, Coakley D (2000). Ocular microtremor (OMT): a new neurophysiological approach to multiple sclerosis. J Neurol Neurosurg Psychiatry.

[CR21] Bolger C, Bojanic S, Sheahan NF, Coakley D, Malone JF (2001). Effect of age on ocular microtremor activity. J Gerontol: Series A.

[CR23] Brown P (1998). A new clinical technique for demonstrating changes in eye acceleration during horizontal saccades in patients with partial internuclear ophthalmoplegias. J Neuroophthalmol.

[CR24] Brown P, Day BL (1997). Eye acceleration during large horizontal saccades in man. Exper Brain Res..

[CR25] Coakley D, Thomas JG (1977). The ocular microtremor record and the prognosis of the unconscious patient. Lancet.

[CR26] De la Torre IM, Hernández Montes MDS, Flores-Moreno JM, Santoyo FM (2016). Laser speckle based digital optical methods in structural mechanics: a review. Opt Las Eng..

[CR27] Discombe RM, Cotterill ST (2015). Eye tracking in sport: A guide for new and aspiring researchers. Sport Exer Psychol Rev.

[CR28] Hannula DE, Althoff RR, Warren DE, Riggs L, Cohen NJ, Ryan JD (2010). Worth a glance: using eye movements to investigate the cognitive neuroscience of memory. Front Hum Neurosci.

[CR29] Heaney M, Kevin LG, Manara AR (2004). Ocular microtremor during general anesthesia: results of a multicenter trial using automated signal analysis. Anesth Analg.

[CR30] Hikosaka O, Takikawa Y, Kawagoe R (2000). Role of the Basal Ganglia in the Control of Purposive Saccadic Eye Movements. Physiol Rev.

[CR31] Kenny E, Coakley D, Boyle G (2013). Ocular microtremor measurement using laser-speckle metrology. J Biomed Opt.

[CR32] Kenny E, Coakley D, Boyle G (2013). Biospeckle in the human sclera and impact on laser speckle correlation measurement of eye tremor. J Biomed Opt.

[CR33] Kenny E, Coakley D, Boyle G (2014). Non-contact in vivo measurement of ocular microtremor using laser speckle correlation metrology. Physiol Meas.

[CR34] Kevin LG, Cunningham AJ, Bolger C (2002). Comparison of ocular microtremor and bispectral index during sevoflurane anaesthesia. Br J Anaesth.

[CR35] Kredel R, Vater C, Klostermann A, Hossner E-J (2017). Eye-tracking technology and the dynamics of natural gaze behavior in sports: A systematic review of 40 years of research. Front Psychol.

[CR36] Lal V, Truong D (2019). Eye movement abnormalities in movement disorders. Clin Parkin Rel Disord..

[CR37] McCamy MB, Collins N, Otero-Millan J (2013). Simultaneous recordings of ocular microtremor and microsaccades with a piezoelectric sensor and a video-oculography system. PEER J..

[CR39] Molitor RJ, Ko PC, Ally BA (2015). Eye movements in Alzheimer's disease. J Alzheimers Dis.

[CR40] Moran A, Campbell M, Ranieri D (2018). Implications of eye tracking technology for applied sport psychology. J Sport Psychol Act..

[CR41] Mucha A, Collins MW, Elbin R (2014). A brief vestibular/ocular motor screening (VOMS) assessment to evaluate concussions: preliminary findings. Am J Sports Med.

[CR01] Page MJ, McKenzie JE, Bossuyt PM, Boutron I, Hoffmann TC, Mulrow CD (2021). The PRISMA 2020 statement: an updated guideline for reporting systematic reviews. BMJ.

[CR42] Ratliff F, Riggs LA (1950). Involuntary motions of the eye during monocular fixation. J Exp Psychol.

[CR43] Robertson J, Timmons S (2007). Non-invasive brainstem monitoring: The ocular microtremor. Art Neurol Res.

[CR44] Ryle JP, Vohnsen B, Sheridan JT (2015). Simultaneous drift, microsaccades, and ocular microtremor measurement from a single noncontact far-field optical sensor. J Biomed Opt.

[CR45] Shakhnovich A (2012) The brain and regulation of eye movement. Springer Science & Business Media.

[CR46] Sheahan NF, Coakley D, Hegarty F, Bolger C, Malone J (1993). Ocular microtremor measurement system: Design and performance. Med Biol Eng Comp.

[CR47] Sheahan NF, Coakley D, Bolger C (1994). Sources of variance in ocular microtremor. Physiol Meas.

[CR48] Spauschus A, Marsden J, Halliday DM, Rosenberg JR, Brown P (1999). The origin of ocular microtremor in man. Exp Brain Res.

[CR49] Stuart S, Hickey A, Vitorio R (2019). Eye-tracker algorithms to detect saccades during static and dynamic tasks: a structured review. Physiol Meas.

[CR50] Stuart S, Lawson RA, Yarnall AJ (2019). Pro-saccades predict cognitive decline in Parkinson's disease: ICICLE-PD. Mov Disord.

[CR51] Terao Y, Fukuda H, Hikosaka O (2017). What do eye movements tell us about patients with neurological disorders? An introduction to saccade recording in the clinical setting. Proce Japan Acad, Series B.

[CR52] Tolosa E, Wenning G, Poewe W (2006). The diagnosis of Parkinson's disease. Lanc Neurol.

[CR53] Wade NJ, Tatler BW, Heller D (2003). Dodge-ing the issue: Dodge, Javal, Hering, and the measurement of saccades in eye-movement research. Perception.

